# Statement of the Number of Patients, and the Diseases under Which They Laboured, Admitted under the Care of the Physicians and Surgeons of the Liverpool General Dispensary in the Year 1805

**Published:** 1806-04

**Authors:** 


*Statement of the Number of Patients, and the Diseases under which they laboured, admitted under the Care of the Physicians and Surgeons of the Liverpool General Dispensary in the Year* 1805.

| | | | |
|---|---|---|---|
| Febres Continuæ - - | 795 | Ophthalmia - - - | 415 |
| ——— Intermittentes | 35 | Gangræna et Sphacelus | 10 |
| Inflammatio - - - | 91 | Cephalalgia et Vertigo | 145 |

Cynanche

| | |
|---|---|
| Cynanche Tonsillaris | 200 |
| —— Trachealis | 15 |
| —— Maligna | 8 |
| Scarlatina | 80 |
| —— Anginosa | 68 |
| Rubeola | 19 |
| Variola | 141 |
| Varricella | 12 |
| Vaccinatio | 822 |
| Erysipelas | 57 |
| Urticaria | 9 |
| Rheumatismus | 656 |
| Pneumonia | 67 |
| Gastritis | 4 |
| Enteritis | 6 |
| Hepatitis | 4 |
| Nephritis | 22 |
| Peritonitis | 3 |
| Aphtha | 110 |
| Epistaxis | 35 |
| Hæmoptysis | 76 |
| Hæmatemesis | 20 |
| Phthisis | 255 |
| Hæmorrhois | 70 |
| Menorrhagia | 97 |
| Leucorrhœa | 63 |
| Blenorrhœa | 14 |
| Hæmaturia | 18 |
| Abortio | 53 |
| Catarrhus | 94 |
| Dysenteria | 173 |
| Cholera et Diarrhœa | 388 |
| Apoplexia | 12 |
| Paralysis | 400 |
| Hemiplegia | 16 |
| Hemicrania | 26 |
| Paraplegia | 5 |
| Syncope | 5 |
| Dyspepsia | 328 |
| Chlorosis | 8 |
| Vomitus | 27 |
| Hypochondriasis | 21 |
| Amenorrhœa | 200 |
| Spasmi | 16 |
| Convulsio | 71 |
| Tetanus | 10 |
| Chorea Sancti Viti | 20 |
| Epilepsia | 57 |
| Palpitatio | 65 |
| Asthma | 174 |
| Tussis et Dyspnœa | 692 |
| Tussis Convulsiva | 125 |
| Colica | 100 |
| —— Pictonum | 20 |
| Diabetes | 20 |
| Hysteria | 151 |
| Melancholia | 2 |
| Insania | 8 |
| Debilitas | 226 |
| Marasmus | 6 |
| Tabes Mesenterica | 4 |
| Hydrops et Anasarca | 233 |
| Ascites | 40 |
| Hydrocephalus | 9 |
| Congesto Capitis | 10 |
| Hydrothorax | 10 |
| Hydrocele | 6 |
| Rachitis | 18 |
| Scrofula | 145 |
| Syphilis et Gonorrhœa | 799 |
| Scorbutus | 21 |
| Icterus | 40 |
| Amaurosis | 16 |
| Dysecœa | 4 |
| Eneuresis | 24 |
| Obstipatio | 280 |
| Nephralgia | 60 |
| Aneurismus | 3 |
| Schirrus et Cancer | 28 |
| Polypus Nasi | 12 |
| Hernia | 85 |
| —— Strangulata | 5 |
| Prolapsus Uteri | 55 |
| —— Ani | 38 |
| Luxatio et Distentio | 85 |
| Amputatio | 9 |
| Vulnus, Contusio et Fractura | 453 |
| Abscessus, Ulcus et Testio | 378 |
| Tumor et Induratio | 138 |
| Curvatura Spinæ | 9 |

Fistula

| | | | |
|---|---|---|---|
| Fistula in Ano | 4 | Calculus Vesicæ | 2 |
| Herpes | 30 | Vermes | 246 |
| Tinea Capitis | 55 | Dentitio | 18 |
| Psora | 835 | | |
| Caries | 10 | | 12,237 |
| Hydrarthus | 10 | | |

